# Incident Signal Power Comparison for Localization of Concurrent Multiple Acoustic Sources

**DOI:** 10.1155/2014/582397

**Published:** 2014-02-20

**Authors:** Daniele Salvati, Sergio Canazza

**Affiliations:** ^1^Department of Mathematics and Computer Science, University of Udine, 33100 Udine, Italy; ^2^Department of Information Engineering, University of Padova, 35131 Padova, Italy

## Abstract

In this paper, a method to solve the localization of concurrent multiple acoustic sources in large open spaces is presented. The problem of the multisource localization in far-field conditions is to correctly associate the direction of arrival (DOA) estimated by a network array system to the same source. The use of systems implementing a Bayesian filter is a traditional approach to address the problem of localization in multisource acoustic scenario. However, in a real noisy open space the acoustic sources are often discontinuous with numerous short-duration events and thus the filtering methods may have difficulty to track the multiple sources. Incident signal power comparison (ISPC) is proposed to compute DOAs association. ISPC is based on identifying the incident signal power (ISP) of the sources on a microphone array using beamforming methods and comparing the ISP between different arrays using spectral distance (SD) measurement techniques. This method solves the ambiguities, due to the presence of simultaneous sources, by identifying sounds through a minimization of an error criterion on SD measures of DOA combinations. The experimental results were conducted in an outdoor real noisy environment and the ISPC performance is reported using different beamforming techniques and SD functions.

## 1. Introduction

The sensory capacity to analyze acoustic space is a very important function of an auditory system. The need for the development of an understanding of the sound environment has attracted many researchers over the past twenty years to build sensory systems that are capable of locating acoustic sources in space. Acoustic source localization (ASL) is an important task in a growing number of applications. Fields of application in which identification of the location of acoustic sources is desired include audio surveillance, teleconferencing systems, hands-free acquisition in car, system monitoring, human-machine interaction, musical control interfaces, videogames, virtual reality systems, voice recognition, fault analysis of machinery, autonomous robots, processors for digital hearing aids, high-quality recording, multiparty telecommunications, dictation systems, and acoustic scene analysis. The aim of an ASL system is to estimate the position of sound sources in space by analyzing the sound field with a microphone array, a set of microphones arranged to capture the spatial information of sound.

Several application areas that may potentially provide advantages in using the acoustic location have led to the development of many signal processing algorithms, which mostly consider the specific acoustic environment, the signal properties, and the localization goal.

ASL can be performed by two basic methods: indirect and direct. The indirect approach is used to estimate source positions by implementing the following two steps: in the first one, a set of time difference of arrivals (TDOAs) are estimated using measurements across various combinations of microphones, and in the second one, when the position of the sensors and the speed of sound are known, the source positions can be estimated using geometric considerations and approximate estimators: closed-formed estimators based on a least squares solution [1–7] (for an overview on closed-form estimators, see [8]) and iterative maximum likelihood estimators [9–15]. The direct approach involves the search space by constructing a spatial energy map and estimating, for each possible point of interest, the values that maximize a specific likelihood function that provides a coherent value from the entire system of arrays. The position of the sources can be estimated directly and spatial likelihood functions can be defined [16–20].

In near-field conditions, since the sources radiate the sound in spherical waves, a hyperboloid describes all of the possible points of an acoustic source that generates the same TDOA to an array of two microphones. Indirect methods aim at estimating TDOAs for microphone pairs, typically using the generalized cross-correlation (GCC) [21] and the adaptive eigenvalue decomposition (AED) [22] based on the blind system identification, which focuses on the impulse responses between the source and the microphones. The extension of the AED in the case of multiple microphones was proposed in [23], and it is efficiently performed with a normalized multichannel frequency-domain least mean square algorithm [24, 25]. However, the steered response power (SRP) is a direct method based on maximizing the power output of a beamformer. Beamforming is a combination of the delayed signals from each microphone in a manner in which an expected pattern of radiation is preferentially observed. In general, the SRP is computed in frequency-domain using the fast Fourier transformer on a signal portion, calculating the response power on each frequency bin, and subsequently fusing these estimates to obtain the final result. The conventional SRP is performed with the delay and sum beamformer [26]; it consists of the synchronization of signals that steer the array in a certain direction, and it sums the signals to estimate the power of the spatial filter. The SRP phase transform (SRP-PHAT) [18] is a widely used filtered beamforming. PHAT filter [21] places equal importance on each frequency by dividing the spectrum by its magnitude. It normalizes the amplitude of the spectral density using only the phase information with the advantage to improve performance in case of moderate noise and reverberation. SRP-PHAT is deeply used due to the fact that it can be efficiently computed by coherent summing the GCC-PHAT from all of the microphone pairs for each possible point of interest. The high-resolution SRP has been developed to improve the performance of the spatial filter, and the adaptive beamformer is called the minimum variance distortionless response (MVDR) due to Capon [27]. The multiple signal classification (MUSIC) algorithm is based on an eigen subspace decomposition method [28, 29], and the estimation of signal parameters via rotational invariance techniques (ESPRIT) is based on subspace decomposition exploiting the rotational invariance [30–32].

In far-field conditions, we are no longer able to detect the spherical wavefront in relationship with the distance of source from an array and the size of the array, and the wavefront is approximate to a plane. In this condition, with an array of microphones, we are able to estimate only the direction of arrival (DOA) of the source but not its distance from the array. In the far-field case the hyperboloid, which is the locus of points that generates the same TDOA to a microphone pair, can be approximated with the cone whose vertex is located at the midpoint of the array. Thus, we need a network of arrays to perform the localization of a source (at least two arrays for two-dimensional space). Hence, the position estimation is computed by intersection of lines and by an approximated solution for overdetermined systems using the linear least squares method. In the case of an array containing *M* microphones (*M* > 2) the DOA estimation can be computed with the indirect method of multichannel cross-correlation coefficient (MCCC) [33, 34], which is based on TDOAs estimation using GCC, and on the use of the spatial prediction error to measure the correlation among multiple signals. It has the advantage of using the redundant information between microphones to estimate the DOA in a more robust manner under a reverberant and noisy condition. The family of SRP direct methods with an array is used to estimate the DOAs of sources by picking the values corresponding to the principal peaks of the steered response power of a beamforming.

Recently, more sophisticated algorithms have been proposed for time delay estimation that use minimum entropy [35, 36] and blind source separation [37–39]. In [39], the authors demonstrate that the broadband independent component analysis methods are more robust against high background noise levels compared with the conventional GCC-PHAT approach.

Both indirect and direct methods have been tested in many single source scenarios; however, in multiple sources cases, they require new considerations. Several works address the problem of multiple sources using a Bayesian approach based on the tracking of the sources and using Kalman filter [40–47] and Particle filter [19, 48–53]. Some studies consider an approach without tracking in reverberant environments [39, 54–57].

In a real open space, the traditional techniques based on Bayesian filters (Kalman and Particle filters) are difficult to apply for localization of concurrent multiple acoustic sources, because sources are often discontinuous with numerous short-duration events and the spatial resolution may be poor in some areas of analysis. Note that in practical applications the localization in a open space needs a reduced number of arrays, due to limited space for installing it and not to invade the monitoring spaces in an excessive way. Besides, methods based on movement tracking can fail in some specific situations: during the initialization phase of the filter, in the presence of sources with unpredictable trajectory (e.g., in the case of rapid changes of the velocity vector), and when two sources have intersecting trajectories.

As a solution to this problem, we present the approach based on the incident signal power comparison (ISPC). A preliminary work was proposed in [58, 59]. This paper describes a detailed step-by-step ISPC algorithm introducing a diagonal loading (DL) [60, 61] for MVDR beamforming, which gives more stable ISP estimation, and reporting new experimental results in a real scenario. ISPC is designed for a distributed array system, and it is based on source extraction and on a verification of similarity among sound sources. The first step consists of source extraction using beamforming techniques and estimation of the incident signal power (ISP) of every source captured on the array. The second step involves the comparison of the ISP spectrum from different arrays using a spectral distance (SD) measure. The ISP spectrum permits identification of sounds so that the spectrum power distance minimizes an error criterion. Therefore, the identification of the correct combination of DOAs is estimated by identifying the minor value of SD measures.

The location in a free-field outdoor environment can be employed for audio surveillance, sound monitoring, and analysis of acoustic scenes. In particular, Section 5 describes a prototype system for multiple source localization in a public space for monitoring a large area with a joint audio-video system, in which the positional estimates by acoustic analysis are used to steer a video-camera consequently.

The paper is organized as follows. After presenting the signal model in Section 2, the multiple sources localization problem is described in Section 3. In Section 4 the ISPC algorithm is presented. Finally, Section 5 illustrates experimental results obtained in a real-world scenario.

## 2. Signal Model

We assume *N* acoustic sources and *R* arrays, each composed of *M* microphones, and consider the omnidirectional characteristics of both the sources and the microphones. We will refer to the model of discrete-time obtained by performing a sampling operation on the continuous-time signal *x*(*t*) with a uniform sampling period *T*_*s*_. A discrete-time signal is expressed by

(1) $$\begin{array}{c} x\left(kT_{S}\right)=x\left(\frac{k}{f_{s}}\right)\;k=0,1,\ldots , \end{array}$$

where *k* is the sample time index and *f*_*s*_ is the sampling frequency. As usual, we will allow the sample period *T*_*s*_ to remain implicit and refer to it simply as *x*(*k*).

The free-field discrete-time signal received by the *m*th microphone of the *r*th array can be modeled as

(2) $$\begin{array}{c} x_{rm}\left(k\right)=\overset{N}{\underset{n=1}{\sum }}\alpha_{rnm}s_{n}\left(k-k_{rn}-\tau_{rnm}\right)+v_{rm}\left(k\right), \end{array}$$

where *α*_*rnm*_ is the attenuation of the sound propagation (inversely proportional to the distance from source *n* to microphone *m* of array *r*), *s*_*n*_(*k*) are the unknown uncorrelated source signals, *k*_*rn*_ is the propagation time from the unknown source *n* to the reference sensor of array *r*, *τ*_*rnm*_ is the TDOA of the signal *n* between the *m*th microphone and the reference of the *r*th array, and *v*_*rm*_(*k*) is the additive noise signal at the sensor *m* of array *r*, assumed to be uncorrelated with not only all of the source signals but also with the noise observed at the other sensors.

In far-field case the relationship between TDOA and DOA can be solved easily with geometrical considerations. Therefore, for a generic pair of microphones with TDOA *τ*_*rn*_, DOA estimate is obtained as

(3) $$\begin{array}{c} \theta_{rn}=\arcsin\left(\frac{\tau_{rn}c}{d}\right), \end{array}$$

where *c* is the speed of sound and *d* the distance between microphones.

The vector Θ_*n*_ for each source *n*, considering the signal model (2), is defined by

(4) $$\begin{array}{c} \Theta_{n}={\left[\theta_{1n},\theta_{2n},\ldots ,\theta_{Rn}\right]}^{T} \end{array}$$

which contains the DOAs of the acoustic source *n* by each array. In the case of *N* sources and *R* arrays, we can write the matrix *R* × *N*, which contains all DOAs of distributed array network as

(5) $$\begin{array}{c} \Theta =\left[\Theta_{1},\Theta_{2},\ldots ,\Theta_{N}\right]=\left[\begin{array}{cccc} \theta_{11} & \theta_{12} & \cdots & \theta_{1N}\\ \theta_{21} & \theta_{22} & \cdots & \theta_{2N}\\ \vdots & \vdots & \ddots & \vdots \\ \theta_{R1} & \theta_{R2} & \cdots & \theta_{RN} \end{array}\right]. \end{array}$$

The estimated DOAs angles, obtained for each array *r*, are written with the following vector:

(6) $$\begin{array}{c} {\underline{\hat{\Theta }}}_{r}=\left[{\hat{\theta }}_{r1},{\hat{\theta }}_{r2},\ldots ,{\hat{\theta }}_{rN}\right], \end{array}$$

where we consider the DOA values in ascending order (${\hat{\theta }}_{r1}<{\hat{\theta }}_{r2}<{\hat{\theta }}_{r3}$, etc.). Next, the estimated sorted DOAs matrix $\underline{\hat{\Theta }}$ is defined as

(7) $$\begin{array}{c} \underline{\hat{\Theta }}=\left[\begin{array}{c} {\underline{\hat{\Theta }}}_{1}\\ {\underline{\hat{\Theta }}}_{2}\\ \vdots \\ {\underline{\hat{\Theta }}}_{R} \end{array}\right]=\left[\begin{array}{cccc} {\hat{\theta }}_{11} & {\hat{\theta }}_{12} & \cdots & {\hat{\theta }}_{1N}\\ {\hat{\theta }}_{21} & {\hat{\theta }}_{22} & \cdots & {\hat{\theta }}_{2N}\\ \vdots & \vdots & \ddots & \ddots \\ {\hat{\theta }}_{R1} & {\hat{\theta }}_{R2} & \cdots & {\hat{\theta }}_{RN} \end{array}\right]. \end{array}$$

The position of the source *n* can be calculated by combining the DOAs estimated by the *R* arrays for that source.

## 3. Multiple Sources Localization

**Figure 1:**
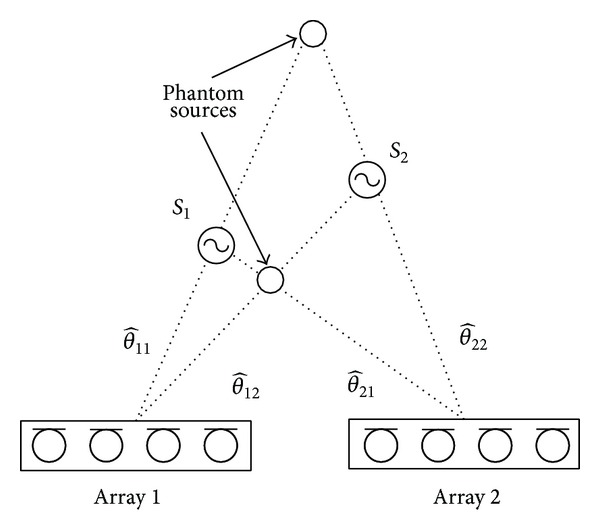
The problem of multiple sources localization.

The multiple sources localization problem is to correctly assign the *R* DOAs values to the source *n*. In some applications, situations arise for which we cannot assign unambiguously TDOAs or DOAs to the same source. The example in Figure 1 shows the case of two sources with a configuration of two arrays for the 2D location. As we can see, the combination of incorrect DOAs leads to an incorrect position estimation. The two DOAs calculated by the two arrays can be combined following two different configurations: (1)  ${\hat{\theta }}_{11}-{\hat{\theta }}_{21}$, ${\hat{\theta }}_{12}-{\hat{\theta }}_{22}$; (2)  ${\hat{\theta }}_{12}-{\hat{\theta }}_{21}$, ${\hat{\theta }}_{11}-{\hat{\theta }}_{22}$. The first configuration implies the correct localization of the sound sources, whereas the second leads to an incorrect localization of both the sources.

In general, the goal is to get the matrix Θ to properly order the values of (7). Considering *θ*_*rn*_ as the *n*th DOA of array *r*, the assignment of the correct value of the DOA for the unknown sources can be ambiguous; namely the exact position of the elements in the matrix of (6) cannot be uniquely determined:

(8) $$\begin{array}{c} {\hat{\theta }}_{rn}⟶\theta_{rn}. \end{array}$$

The possible combinations of the DOAs of matrix (7) are *O* = (*N*!)^(*R*−1)^.

## 4. Incident Signal Power Comparison (ISPC)

Incident signal power comparison (ISPC) combines the DOAs from different arrays by considering the similarity criterion among acoustic sources. To check for this similarity, we can estimate for each array the ISP referring to all estimated DOAs using beamforming techniques. Once the ISPs are obtained, we can define an efficient error criterion for comparing the different possible combinations of the DOAs using a SD measure of ISPs pair between different arrays.

**Figure 2:**
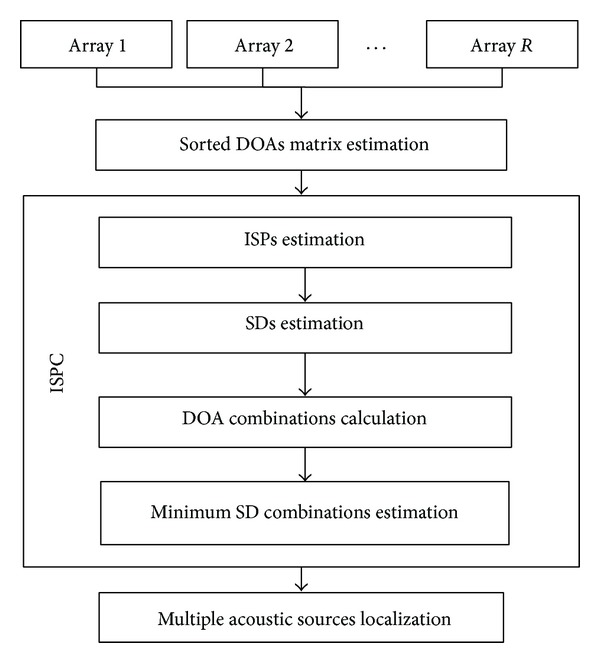
The steps for the ISPC algorithm.

DOA estimation is a crucial step of ASL systems. In a free-field environment for far-field cases, it can be calculated by means of MCCC and SRP methods. After obtaining an estimation of the sorted DOAs matrix (the matrix $\underline{\hat{\Theta }}$ of (7)), the steps of the ISPC algorithm are (1) source extraction using beamforming techniques and estimation of ISPs for each DOA, (2) ISPC using SD measurement between ISPs of different array, (3) calculation of all DOAs combinations, and (4) verification of the most consistent target combinations minimizing an error criterion on SD measurements. Finally, the localization of multiple sources can be computed by considering the estimated DOAs combination.  Figure 2 illustrates the ISPC steps.

### 4.1. Incident Signal Power Estimation

The ISP is the power spectral density of the beamformer output that is steered to a specified direction. The SRP is based on maximizing the power output of a beamformer. Beamforming is a multichannel signal processing techniques that enhance the acoustic signals coming from a specific steered position, while reducing the signals coming from other directions. In the frequency domain, the output of a generic beamformer of *r*th array in matrix notation can be written as

(9) $$\begin{array}{c} Y_{r}\left(f\right)=W^{H}\left(f,\theta_{rn}\right)X_{r}\left(f\right), \end{array}$$

where **X**_*r*_(*f*) = [*X*_*r*1_(*f*), *X*_*r*2_(*f*),…, *X*_*rM*_(*f*)]^*T*^, *Y*_*r*_(*f*) and *X*_*rm*_(*f*) are the discrete Fourier transform of the signals, **W**(*f*, *θ*_*rn*_) = [*W*_1_(*f*, *θ*_*rn*_), *W*_2_(*f*, *θ*_*rn*_),…, *W*_*M*_(*f*, *θ*_*rn*_)]^*T*^ is the vector of the beamformer weights for steering and filtering the data on the direction *θ*_*rn*_, *f* is the frequency bin index, and the superscript *H* represents the Hermitian (complex conjugate) transpose.

The ISP of the beamformer output for a generic frequency *f* is given by

(10) ISP(f)=E{|Yr(f)|2}=WH(f,θrn)E{Xr(f)XrH(f)}W(f,θrn)=WH(f,θrn)Φr(f)W(f,θrn),

where Φ_*r*_(*f*) is the cross-spectral density matrix, which is square *M* × *M* and symmetric, and *E*{·} denotes mathematical expectation. We consider a power spectrum calculated with *f* = *F*_min⁡_, *F*_min⁡_ + 1,…, *F*_max⁡_, where *F*_min⁡_ and *F*_max⁡_ are the index values of a specific frequency range (FR), which defines the range interesting for the optimal performance of the ISPC algorithm. Note that beamformer pattern function is frequency dependent; then the main lobe narrows with increasing frequency and spatial aliasing can occur (for a comprehensive dissertation, refer to [62]).

Several beamforming techniques exist (a review can be found in [63]); however, the spatial filter methods that are used for comparing ISPC experimental results are the SRP based on delay and sum beamforming, the SRP with the Dolph-Chebyshev window (SRP-DC), and the MVDR with DL.

Hence, the ISP corresponding to delay and sum SRP can be written from (10) as

(11) $$\begin{array}{c} \text{IS}{\text{P}}_{rn}^{\text{SRP}}\left(f\right)=A^{H}\left(f,\theta_{rn}\right)\Phi_{r}\left(f\right)A\left(f,\theta_{rn}\right), \end{array}$$

where **A**(*f*, *θ*_*rn*_) is the steering vector corresponding to direction *θ*_*rn*_. For a uniform linear array with microphone distance *d*, the steering vector takes the form

(12) A(f,θrn)=[1,ej2π(f−1)dfssinθrn/c,…,e(j2π(f−1)dfssinθrn/c)(M−1)]T.

The SRP-DC is obtained from (11) introducing the Dolph-Chebyshev window **w**:

(13) $$\begin{array}{c} \text{IS}{\text{P}}_{rn}^{\text{SRP-DC}}\left(f\right)={\left[w⊙A\left(f,\theta_{rn}\right)\right]}^{H}\Phi_{r}\left(f\right)\left[w⊙A\left(f,\theta_{rn}\right)\right], \end{array}$$

where ⊙ denotes element-wise multiplication.

The adaptive MVDR beamforming [27] is based on minimization problem of the following equation

(14) argminW(f,θrn) WH(f,θrn)Φr(f)W(f,θrn)subject  to WH(f,θrn)A(f,θrn)=1.

The aim of the MVDR filter is to minimize the noise and sources coming from different directions, while keeping a fixed gain on the desired direction. Solving (14) using the method of Lagrange multipliers, we can write

(15) $$\begin{array}{c} W_{\text{MVDR}}\left(f,\theta_{rn}\right)=\frac{\Phi_{r}^{-1}\left(f\right)A\left(f,\theta_{rn}\right)}{A^{H}\left(f,\theta_{rn}\right)\Phi_{r}^{-1}\left(f\right)A\left(f,\theta_{rn}\right)}. \end{array}$$

In practical applications, the inverse of the cross-spectral density matrix can be calculated using the Moore-Penrose pseudoinverse, defined as

(16) $$\begin{array}{c} \Gamma^{+}=VS^{-1}U^{H}, \end{array}$$

where Γ = **U** **S** **V** ^*H*^ is the singular value decomposition of the matrix Γ. Moreover, if the cross-spectral density matrix is ill-conditioned, the spatial spectrum may not exist. Therefore, a DL [60, 61] method is adopted to calculate the inverse matrix in a stable way. The ISP with MVDR filter and DL becomes

(17) $$\begin{array}{c} \text{IS}{\text{P}}_{rn}^{\text{MVDR}}\left(f\right)=\frac{1}{A^{H}\left(f,\theta_{rn}\right){\left(\Phi_{r}\left(f\right)+\mu I\right)}^{+}A\left(f,\theta_{rn}\right)}, \end{array}$$

where **I** is the identity matrix and *μ* is the loading level:

(18) $$\begin{array}{c} \mu =\frac{1}{L}\mathrm{tr}\left\{\Phi_{r}\left(f\right)\right\}\Delta , \end{array}$$

where tr⁡{·} denotes the trace of the squared matrix and Δ is the normalized loading constant. Typically, the values are Δ = 0.1, Δ = 1, Δ = 10 [64].

Therefore, we can define the matrix **P** containing all the ISPs related to the matrix (7):

(19) P=[P11,P12,…,P1N,P21,P22,…,P2N,PR1,PR2,…,PRN]

which has a dimension of (*F*_max⁡_ − *F*_min⁡_) × *RN*, where the total number of ISPs is *I* = *NR* and **P**_*rn*_ = [ISP_*rn*_(*F*_min⁡_), ISP_*rn*_(*F*_min⁡_ + 1),…, ISP_*rn*_(*F*_max⁡_)]^*T*^.

### 4.2. Spectral Distance Estimation

To compare the ISPs of different arrays, spectral distance (SD) functions are used. Distance measures produce measurements of the dissimilarity of two sound spectra. We define the SD estimation between the ISP_*rn*_ and the ISP_*ij*_ of two DOAs of different arrays as

(20) $$\begin{array}{c} E_{rnij}=\frac{1}{L}\overset{F_{\max}}{\underset{f=F_{\min}}{\sum }}\left|𝒮\left\{\text{IS}{\text{P}}_{rn}\left(f\right),\text{IS}{\text{P}}_{ij}\left(f\right)\right\}\right|, \end{array}$$

where *L* = (*F*_max⁡_ − *F*_min⁡_ + 1), *r* and *i* are the index labels of the array, *r* ≠ *i*, *n* and *j* are the index labels for the sorted DOAs of array, and *𝒮*{ISP_*rn*_(*f*), ISP_*ij*_(*f*)} is the SD function to measure the dissimilarity of spectra. We consider the four most common SD functions to verify how our system performance varies as a function of different equations. A classic spectral estimation method is linear prediction (LP) [65], for which we insert a negative one to standardize the minimum to zero as all functions

(21) $$\begin{array}{c} E_{rnij}^{\text{LP}}=\frac{1}{L}\overset{F_{\max}}{\underset{f=F_{\min}}{\sum }}\left|\frac{\text{IS}{\text{P}}_{rn}\left(f\right)}{\text{IS}{\text{P}}_{ij}\left(f\right)}-1\right|. \end{array}$$

The other functions are the Itakura-Saito (IS) distance measure [66]

(22) $$\begin{array}{c} E_{rnij}^{\text{IS}}=\frac{1}{L}\overset{F_{\max}}{\underset{f=F_{\min}}{\sum }}\left|\frac{\text{IS}{\text{P}}_{rn}\left(f\right)}{\text{IS}{\text{P}}_{ij}\left(f\right)}-\log\frac{\text{IS}{\text{P}}_{rn}\left(f\right)}{\text{IS}{\text{P}}_{ij}\left(f\right)}-1\right|, \end{array}$$

the root mean square (RMS) log [67]

(23) $$\begin{array}{c} E_{rnij}^{\text{RMS}}=\frac{1}{L}\overset{F_{\max}}{\underset{f=F_{\min}}{\sum }}{\left(\log\frac{\text{IS}{\text{P}}_{rn}\left(f\right)}{\text{IS}{\text{P}}_{ij}\left(f\right)}\right)}^{2}, \end{array}$$

and the COSH measure [68]

(24) ErnijCOSH=1L∑f=Fmin⁡Fmax⁡|ISPrn(f)ISPij(f)−log⁡ISPrn(f)ISPij(f)+ISPij(f)ISPrn(f)−log⁡ISPij(f)ISPrn(f)−2|.

The total number of SD measures between all the ISPs pair of different arrays is *Q* = *N* ^2^ *R*(*R* − 1)/2.

### 4.3. DOA Combinations Calculation

**Figure 3:**
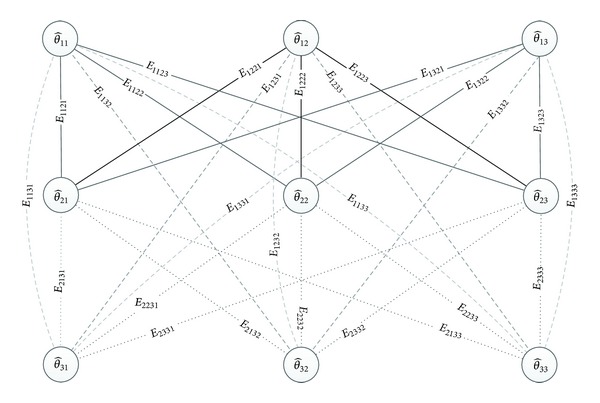
Graphic representation of DOAs and SDs estimations.

Let us represent the sorted matrix of the DOAs using the graph theory to better understand the DOAs combinations calculation and the verification of the most consistent target combination minimizing an error criterion. Then, we can express the matrix (7) and all of its combinations as being composed of nodes and edges, connecting pairs of vertices. An example of three arrays and three sources is shown in Figure 3. Each row of the graph contains the sorted DOAs of an array: ${\underline{\hat{\Theta }}}_{1}={\left[{\hat{\theta }}_{11},{\hat{\theta }}_{12},{\hat{\theta }}_{13}\right]}^{T}$, ${\underline{\hat{\Theta }}}_{2}={\left[{\hat{\theta }}_{21},{\hat{\theta }}_{22},{\hat{\theta }}_{23}\right]}^{T}$, and ${\underline{\hat{\Theta }}}_{3}={\left[{\hat{\theta }}_{31},{\hat{\theta }}_{32},\ldots ,{\hat{\theta }}_{3N}\right]}^{T}$. Each DOA is a node of graph and the edges represent the possible connections between nodes with the values *E*_*rnij*_, which is the estimated SD between the ISPs on array *r* of DOA *i* and on array *n* of DOA *j*. The combination of incorrect DOAs leads to an incorrect position estimation (see Figure 1). Thus, if we represent a combination of DOAs as a sum of values of the edges that connect the nodes, we expect that the minimum value of different sums corresponds to the correct combination. To calculate the possible combinations of DOAs between the arrays, it is helpful to introduce a matrix labeling of DOAs (7):

(25) $$\begin{array}{c} B=\left[\begin{array}{cccc} B_{11} & B_{12} & \ldots & B_{1N}\\ B_{21} & B_{22} & \ldots & B_{2N}\\ \vdots & \vdots & \ddots & \vdots \\ B_{R1} & B_{R2} & \ldots & B_{RN} \end{array}\right] \end{array}$$

in which the generic element is expressed as

(26) $$\begin{array}{c} B_{rn}=\left(r-1\right)N+n \end{array}$$

with *r* = 1,2,…, *R* and *n* = 1,2,…, *N*. The matrix label **B** associates the position of the DOAs referring to the sorted matrix $\underline{\hat{\Theta }}$. Estimating the minimum error of an SD combination, we can obtain the matrix $\hat{\Theta }$ with the correct position of the DOAs, in which each column contains the DOAs of the source *n*.

Furthermore, we can represent the graph representation of DOAs and the SDs as the adjacency matrix Λ, which is an *RN* × *RN* matrix of SD values. The entry in row (*B*_*rn*_ = 1,…, *RN*) and column (*B*_*ij*_ = 1,…, *RN*) is defined as an SD estimation *E*_*rnij*_ if there is an edge connecting vertex *B*_*rn*_ and vertex *B*_*ij*_ in the graph, or it is defined as zero otherwise. The relationships between DOAs and SDs can be expressed by the following equation of the adjacency matrix element:

(27) $$\begin{array}{c} \Lambda_{B_{rn}B_{ij}}=E_{rnij}. \end{array}$$

The symmetric adjacency matrix results in the following equation:

(28) $$\begin{array}{c} \Lambda =\left[\begin{array}{cccccccccc} 0 & \ldots & 0 & E_{1121} & \ldots & E_{112N} & \ldots & E_{11R1} & \ldots & E_{11RN}\\ \vdots & \ddots & \vdots & \vdots & \ddots & \vdots & \ddots & \vdots & \ddots & \vdots \\ 0 & \ldots & 0 & E_{1N21} & \ldots & E_{1N2N} & \ldots & E_{1NR1} & \ldots & E_{1NRN}\\ E_{2111} & \ldots & E_{211N} & 0 & \ldots & 0 & \ldots & E_{21R1} & \ldots & E_{21RN}\\ \vdots & \ddots & \vdots & \vdots & \ddots & \vdots & \ddots & \vdots & \ddots & \vdots \\ E_{2N11} & \ldots & E_{2N1N} & 0 & \ldots & 0 & \ldots & E_{2NR1} & \ldots & E_{2NRN}\\ \vdots & \ddots & \vdots & \vdots & \ddots & \vdots & \ddots & \vdots & \ddots & \vdots \\ E_{R111} & \ldots & E_{R11N} & E_{R121} & \ldots & E_{1121} & \ldots & 0 & \ldots & 0\\ \vdots & \ddots & \vdots & \vdots & \ddots & \vdots & \ddots & \vdots & \ddots & \vdots \\ E_{RN11} & \ldots & E_{RN1N} & E_{RN21} & \ldots & E_{RN2N} & \ldots & 0 & \ldots & 0 \end{array}\right]. \end{array}$$

These SD values are weights of the edges of the graph. An example of three arrays and three sources is presented in Figure 3; in this example, we have 27 total SD comparisons (3 for each source).

To calculate all possible combinations of DOAs, we can work on the label matrix **B**. Considering that the first row of **B** related to the first array remains unchanged, we can compute the combinations in two steps. In the first step, the permutations of the *N* labels of **B** for each *R* − 1 row (for each array) are calculated. The number of permutations for each row is *U* = *N*!. Thus, we define the permutation matrix *T* as

(29) T=[T1,T21,T22,…,T2U,T31,T32,…,T3U,…,TR1,TR2,…,TRU],

where

(30) $$\begin{array}{c} T_{1}={\left[B_{11},B_{12},\ldots ,B_{1N}\right]}^{T}, \end{array}$$

(31) $$\begin{array}{c} T_{ru}={𝒫}_{u}\left\{{\left[B_{r1},B_{r2},\ldots ,B_{rN}\right]}^{T}\right\}, \end{array}$$

where **T**_*ru*_ is the vector of *u*th permutation *𝒫*_*u*_ (*u* = 1,…, *U*) of *r*th array (*r* = 2,3,…, *R*), which contains the *N* DOAs label of row *r*. The matrix **T** has a dimension of *N* × *U*(*R* − 1) + 1. In the second step, the combinations of column indices of matrix **T** with value from 2 to *RU* give the *U* ^(*R*−1)^ = *N*!^(*R*−1)^ = *O* possible combinations. We consider the combinations of *R* − 1 groups, each one composed by *U* elements (the permutation), assuming that one member (the index column of matrix **T**) from each of the *R* − 1 groups is used in each combination and assuming that the order is not a distinguishing factor. We define a matrix **Z** of dimension *O* × (*R* − 1), which stores the combinations of groups of column indices of matrix **T**:

(32) $$\begin{array}{c} Z=\left[\begin{array}{cccc} Z_{11} & Z_{12} & \ldots & Z_{1(R-1)}\\ Z_{21} & Z_{22} & \ldots & Z_{2(R-1)}\\ \vdots & \vdots & \ddots & \vdots \\ Z_{O1} & Z_{O2} & \ldots & Z_{O(R-1)} \end{array}\right]. \end{array}$$

The generic element *Z*_*or*_ with *o* = 1,2,…, *O* and *r* = 1,2,…, *R* − 1 can be calculated with the following equations:

(33) $$\begin{array}{c} Z_{(o+i-1)r}=U\left(r-1\right)+2,\;i=1,2,\ldots ,U_{1},\\ Z_{(o+U_{1}+1)r}=\{\begin{array}{cc} Z_{(o+U_{1}+1)r}, & \text{if}\;\;Z_{(U_{1}+1)r}>U_{2},\\ Z_{(o+U_{1}+1)r}+1, & \text{otherwise}, \end{array} \end{array}$$

where *U*_1_ = *U* ^(*r*−1)^ and *U*_2_ = *U*(*r* − 1) + *U* + 1.

Hence, a combination label matrix **C** of *I* × *O* dimension is used to store the DOA label of all combinations:

(34) $$\begin{array}{c} C=\left[C_{1},C_{2},\ldots ,C_{O}\right], \end{array}$$

where **C**_*o*_ is the vector, which contains the *I* DOA labels of combination *o*:

(35) Co=[B11,TZo11,TZo21…,TZo(R−1)1,B12,TZo12,TZo22,…,TZo(R−1)2,…,B1N,TZo1N,TZo2N,…,TZo(R−1)N]T=[C1o,C2o,…,CIo]T.

### 4.4. Minimum SD Measure Estimator

For each source, identified by *R* nodes (the arrays), we have *R*(*R* − 1)/2 edges; then, the number of edges for a combination of DOAs is *G* = *NR*(*R* − 1)/2 = *Q*/*N*. The values of matrix **C** are used to calculate the SD estimation of all combinations. Thus, we can define the SD estimation of the generic combination *o* as the sum of the weights of all the edges:

(36) $$\begin{array}{c} D_{o}=\overset{N}{\underset{n=1}{\sum }}\;\overset{R-1}{\underset{r=1}{\sum }}\;\overset{R-r}{\underset{i=1}{\sum }}\Lambda_{C_{o_{1}o}C_{o_{2}o}}, \end{array}$$

where *o* = 1,2,…, *O*, *o*_1_ = (*n* − 1)*R* + *r* and *o*_2_ = (*n* − 1)*R* + *r* + *i*. Accordingly, we define the SD vector of all combinations

(37) $$\begin{array}{c} D={\left[D_{1},D_{2},\ldots ,D_{O}\right]}^{T}. \end{array}$$

Finally, the index of the minimum value of the vector **D** identifies the target combination as

(38) $$\begin{array}{c} \hat{o}=\underset{o}{\text{argmin}}\;D_{o}, \end{array}$$

and the DOAs matrix $\hat{\Theta }$ is estimated by ordering the label matrix **B** with the combination $C_{\hat{o}}$.

### 4.5. Overall Procedure

The processing steps of the full ISPC algorithm are summarized in Algorithm 1. After the DOAs estimation and creation of the matrix $\underline{\hat{\Theta }}$ defined by (7) the ISPC algorithm is applied if multiple sources are detected. In practice, the matrix does not always present all the DOA values. In these cases, the missing value of array *r* can be represented with a zero value in the label matrix **B** (25). The overall procedure is composed by the following steps: (1) building of the label matrix **B** (25) and calculation of ISPs and the matrix **P** (19); (2) estimation of the SD measurements between all ISP pairs of arrays and creation of the adjacency matrix Λ (28); (3) calculation of the permutations matrix **T** (29) and the all DOA combination matrix **C** (34); (4) calculation of the vector **D** (37) that contains the SD estimation for each DOAs combination and finding the minimum value of **D** (38), for using the index value $\hat{o}$ in the matrix **C** to properly order the matrix $\underline{\hat{\Theta }}$ and estimate the matrix $\hat{\Theta }$.

## 5. Experimental Results

**Figure 4:**
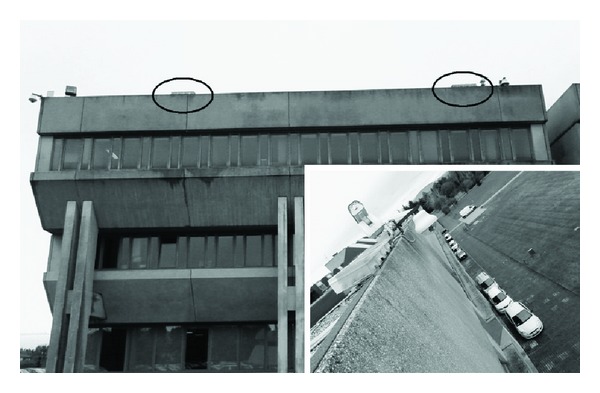
The prototype installed on the roof of the University building. The two arrays are encircled.

The experimental results were conducted in an outdoor real noisy environment and the ISPC performance is reported using different beamforming techniques (SRP, SRP-DC, MVDR) and SD estimations (LP, IS, RMS, COSH). A prototype system for two-dimensional localization has been installed on the roof of the building that houses the Computer Science Department in Udine University (Figure 4).

### 5.1. System Setup

**Figure 5:**
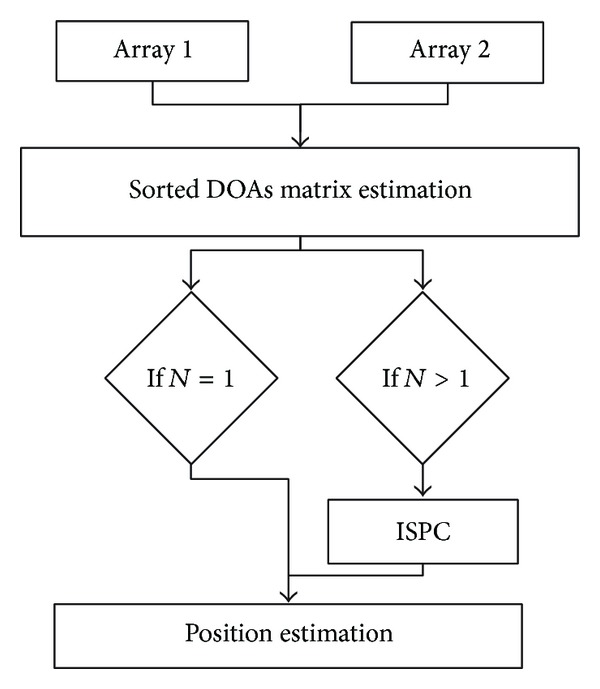
The block diagram of the processor showing the data flow of all of the tasks of the experimental prototype.

The acoustic localization prototype includes two linear arrays, each composed of four omnidirectional microphones. Very small sized arrays are used because a real application of such systems would require that the public spaces are not invaded in an excessive way; therefore, there might not be enough space to install the arrays. The arrays are located 11.4 m apart at a height of 12.1 m above the plane. The sample rate of the digital system is 48 kHz, and the microphone distance is 25 cm. The system consists of two parallel processing lines, corresponding to the Array 1 and Array 2 (Figure 5).

The first processing step is the DOAs estimation. SRP-PHAT is used for the DOAs estimation. The values corresponding to the principal *N* peaks of the SRP-PHAT function (in practice, those peaks which are above a given threshold) allow the DOAs estimation of the *N* acoustic sources. The assumed DOA range is −90° +90°, where zero is in front of the array and the microphone reference is the first from left.

**Figure 6:**
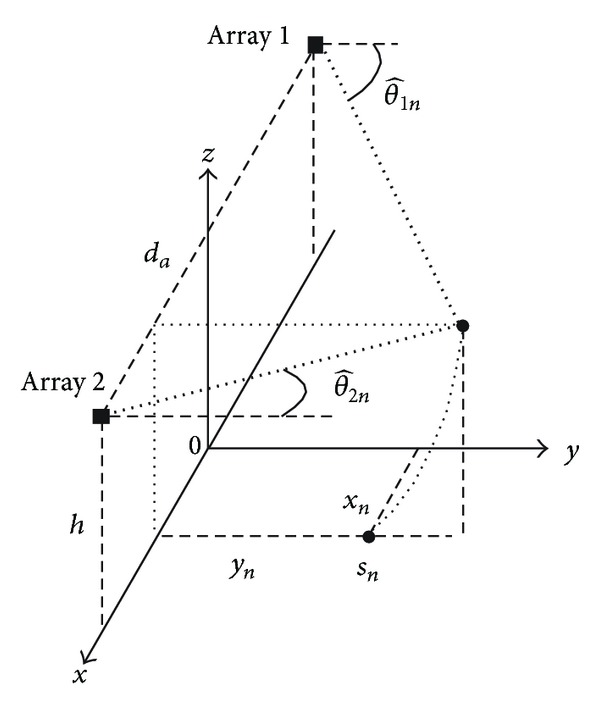
The two-dimensional position of the source of the experimental prototype.

In the second step, the two-dimensional coordinates of the source can be estimated by combining the DOAs at the arrays. If more than one source is identified, a beamformer and an SD comparison provide a guide to solve the problem of associating the DOAs of the Array 1 with those of the Array 2. The calculation of the two-dimensional position of the source is a simple triangulation problem. However, we must consider that the two arrays are not coincidental with the plane of interest but are placed at a certain height. We must consider that the possible points identified by the DOA are located on a cone surface whose vertex is placed in the array and whose axis is the straight line joining the two arrays. Every array represents a cone: the intersection of the two cones is represented by a circumference. The intersection point between the circumference and the plane of interest is the estimation of the source distance from arrays (see Figure 6). Hence, we consider *d*_*a*_ to be the distance of the arrays, *h* to be the height of arrays above the plane of interest, and ${\hat{\theta }}_{1n}$ and ${\hat{\theta }}_{2n}$ to be the DOA estimated on the Array 1 and Array 2, and we obtain

(39) $$\begin{array}{c} x_{n}=\frac{d_{a}}{2}\left(\frac{\tan{\hat{\theta }}_{2n}+\tan{\hat{\theta }}_{1n}}{\tan{\hat{\theta }}_{2n}-\tan{\hat{\theta }}_{1n}}\right),\\ y_{n}=\sqrt{{\left(\frac{d_{a}}{\tan{\hat{\theta }}_{2n}-\tan{\hat{\theta }}_{1n}}\right)}^{2}-h^{2}}. \end{array}$$

**Figure 7:**
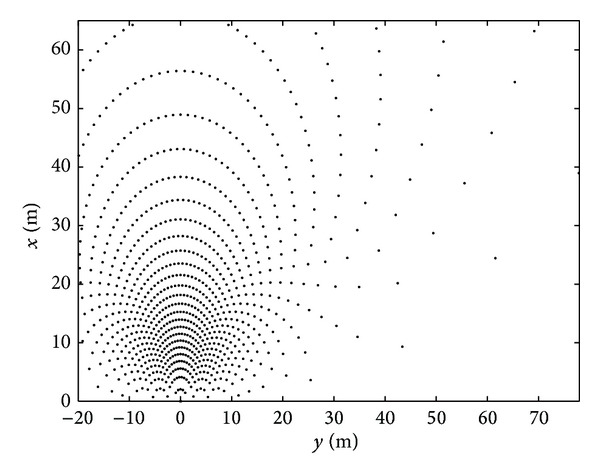
The *x*-*y* sample space position of the plane of interest.

The spatial resolution of the system depends on the distance between the microphones, the distance between the arrays, and the sample frequency of digital system.  Figure 7 shows the possible *xy* coordinates of the considered area of analysis. The zero of the *xy* axes reference is located in the middle of the distance between the two arrays. The spatial resolution tends to decrease with an increasing distance from the arrays and an increasing angle from the axis perpendicular to the array.

### 5.2. Experiment Setup

**Figure 8:**
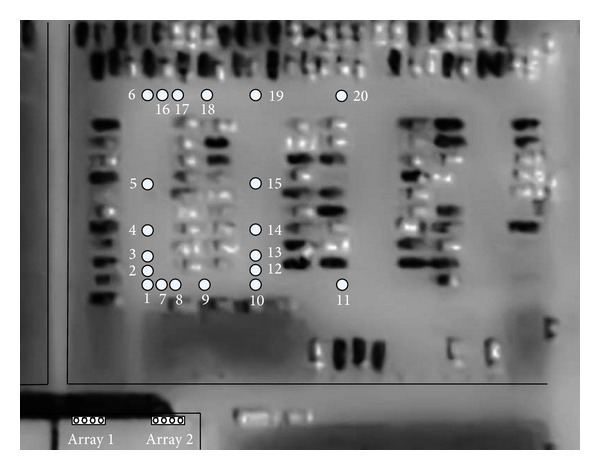
Map of the study area indicating the position of arrays (Array 1 and Array 2) and sources (the twenty labeling numbers: 1,2,…, 20).

Experiments were conducted that consider the area of analysis of 60 × 90 m shown in Figure 8, that is, the parking lots of the University. Twenty zones of acoustic source positioning are considered. They are labeled with a number as we can see in Figure 8. The sources used are a human voice (*s*_1_), a hammer striking an iron bar (*s*_2_), a motor car (*s*_3_), and a honk car (*s*_4_). The hammer striking an iron bar and the honk car are short-duration event sounds.

Two types of experiments were performed. The first type used sounds with different spectral content, named Test 1. The second type, instead, used sounds with similar spectral content, named Test 2. Test 1 is composed of thirty-two parts (*p*_1_, *p*_2_,…, *p*_32_), each one with three sources placed in different positions (see Table 1). In various parts of Test 1, the sources were positioned at increasing distances along the *y* axis and the *x* axis. Table 1 also reported the sound pressure level (SPL) of each source. The environmental noise was in a range of 40–50 dB(A). In Test 2, two car sounds were used. The test was performed by placing two car sources in 1 and 7, as shown in Figure 8.

### 5.3. System Localization Evaluation

An evaluation of the system localization using a single source for each position was computed.  Table 2 shows the real *xy* coordinates of the source points and the root mean square (RMS) error of the estimation using SRP-PHAT method. We can see that the estimation error increases in distant areas and when the angle of incidence on the array is large.

### 5.4. ISPC Evaluation

Tables 3 and 4 summarize the results for Test 1 and Test 2, respectively, comparing the localization success rate (as a percentage) with different beamforming algorithms (SRP, SRP-DC, and MVDR) and SD functions (LP, IS, RMS, and COSH).

The localization success rate is the ratio between the number of correct combinations and the number of ambiguities (NOA). NOA is the number of frames in which we have ambiguity to properly associate the DOAs to the sources; that is, the associations are incorrect in practice. The audio signal frame was divided into 17.5 ms overlapping and a Hann-windowed with a length of 140 ms. The parking area, where the tests were conducted, is a public area. Thus, we must consider that there are other sources in the acoustic scene: other sounds of cars that are moving in the parking area and in the nearby streets.

Table 3 summarizes the results of all thirty-two tests (Test 1). The number of NOA is 750, and the three frequency ranges (FR) for the SD estimation are 20–675 Hz, 20–2000 Hz, and 20–8000 Hz. The frequency value of 675 Hz takes into account the spatial aliasing limit, which, in our case, is *f* = *c*/(2*d*) = 337/(2 · 0.25) = 675 Hz. The phenomena of spatial aliasing implies that the main lobe of the beamformer has a set of identical copies, called grating lobes. The appearance of grating lobes is a function of both microphone spacing and incident frequency. When fully visible, a grating lobe is equal in amplitude to the main lobe of the array. This fact reduces the array response, and, therefore, by defining the spatial sampling requirement and removing the grating lobes, we obtain a greater efficiency in the ISPC.

Table 4 depicts the results of Test 2 with an FR of 20–675 Hz and a NAM of 100. We can note that the accuracy decreases, especially with regard to the RMS and COSH functions, and this result highlights the limitation of the proposed approach in the case of spectrally similar sources.

The best results for Test 1 were obtained with the RMS log SD function and FR = [20–675] Hz. MVDR has the greatest capacity for location with 90.1% of successful DOAs association.

## 6. Conclusions

The novel incident signal power comparison algorithm is used to solve the ambiguous problem of correctly linking the DOAs from different arrays to the same source in a far-field condition with concurrent sources. Experimental results have shown that this approach can be a solution for a multisource localization that requires a frame-to-frame analysis, that is, in those cases in which the traditional filtering approach can not be applied. An evaluation of the system in a real scenario is reported, installing a hardware/software prototype on the roof of the University building and analyzing the results comparing three types of beamforming and four functions for the SD estimation. The interest in locating in a far-field outdoor context may be attractive for audio surveillance, sound monitoring, and the analysis of acoustic scenes. The ISPC is successfully used in a joint audio-video system for monitoring a large area. The best performances are obtained with RMS SD measure on frequency range between 20 Hz and the spatial aliasing frequency limit. We achieved a success rate of 90.1% using MVDR beamforming. We showed the limitation of the proposed algorithm in case of sources that have a similar spectral content.

## Figures and Tables

**Algorithm 1:**
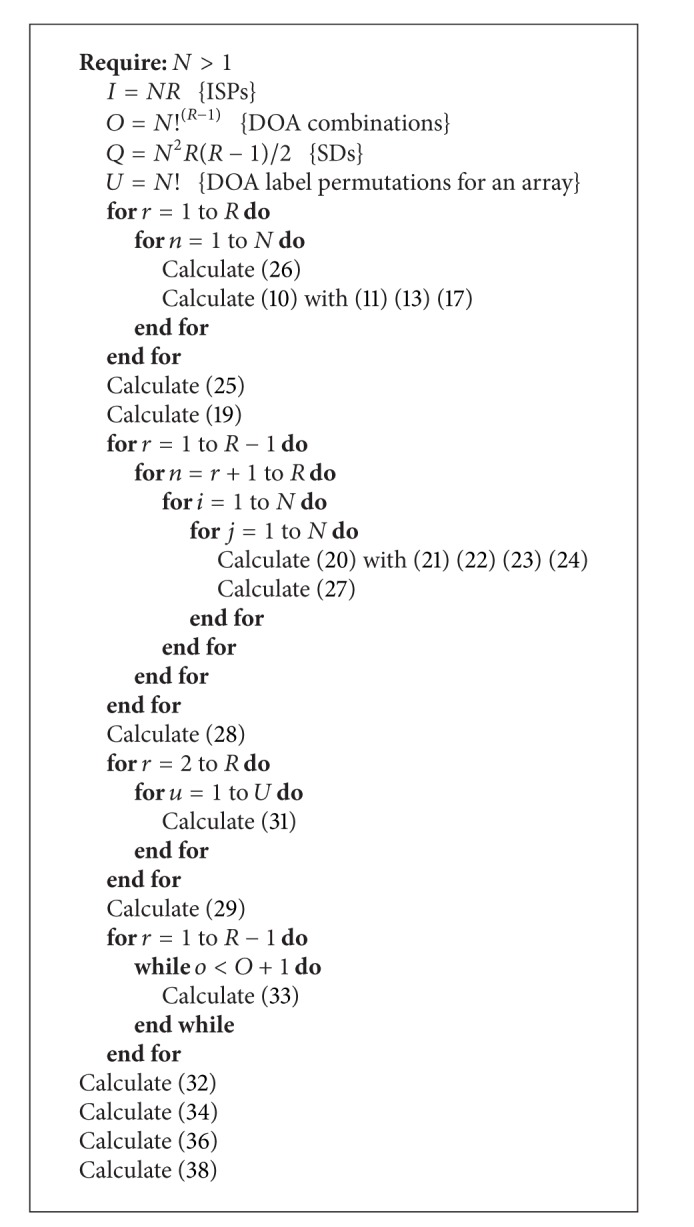
ISPC.

**Table 1:** Position reporting the source label for each test and the SPL of the sources.

Test label	*s*_1_ (Voice)	*s*_2_ (Hammer)	*s*_3_ (Motor car)	*s*_4_ (Honk)
	70 dB(A)	100 dB(A)	68 dB(A)	88 dB(A)
*p*_1_	1	2	3	—
*p*_2_	1	3	4	—
*p*_3_	1	4	5	—
*p*_4_	1	5	6	—
*p*_5_	1	7	8	—
*p*_6_	1	8	9	—
*p*_7_	1	9	10	—
*p*_8_	1	10	11	—
*p*_9_	2	3	1	—
*p*_10_	3	4	1	—
*p*_11_	4	5	1	—
*p*_12_	5	6	1	—
*p*_13_	7	7	1	—
*p*_14_	8	8	1	—
*p*_15_	9	10	1	—
*p*_16_	10	11	1	—
*p*_17_	10	12	—	13
*p*_18_	10	13	—	14
*p*_19_	10	14	—	15
*p*_20_	10	15	—	19
*p*_21_	6	16	—	17
*p*_22_	6	17	—	18
*p*_23_	6	18	—	19
*p*_24_	6	19	—	20
*p*_25_	12	13	—	10
*p*_26_	13	14	—	10
*p*_27_	14	15	—	10
*p*_28_	15	18	—	10
*p*_29_	16	17	—	6
*p*_30_	17	18	—	6
*p*_31_	18	19	—	6
*p*_32_	19	20	—	6

**Table 2:** Position referring to Figure 8 and the RMS errors of the localization estimation using SRP-PHAT method.

Source label	*x* (m)	*y* (m)	RMS error
1	1.5	20	1.1
2	1.5	23	1.7
3	1.5	26	2.2
4	1.5	32	1.9
5	1.5	38	2.5
6	1.5	52	4.1
7	4.5	20	0.9
8	7.5	20	4.2
9	10.5	20	8.6
10	20	20	18.2
11	30	20	8.8
12	20	23	9.2
13	20	26	15.8
14	20	32	15.7
15	20	38	4.8
16	4.5	52	4.2
17	7.5	52	8.3
18	10.5	52	17.5
19	20	52	20.2
20	30	52	23.6

**Table 3:** Results of Test 1 reporting the summary of the thirty-two tests (*p*_1_, *p*_2_,…, *p*_32_).

(Hz)	Localization success rate (%)											
FR	SRP-LP	SRP-IS	SRP-RMS	SRP-COSH	SRP-DC-LP	SRP-DC-IS	SRP-DC-RMS	SRP-DC-COSH	MVDR-LP	MVDR-IS	MVDR-RMS	MVDR-COSH
20–675	28.4	38.2	85.2	79.1	23.4	37.8	86.4	73.7	43.6	68.6	90.1	83.4
20–2000	42.1	45.2	72.3	63.5	43.8	42.4	71.2	61.1	45.4	63.1	77.6	72.1
20–8000	53.2	52.4	69.5	58.5	51.9	49.4	68.3	58.7	48.4	56.0	65.5	61.8

**Table 4:** Results of Test 2 using two car sounds.

(Hz)	Localization success rate (%)											
FR	SRP-LP	SRP-IS	SRP-RMS	SRP-COSH	SRP-DC-LP	SRP-DC-IS	SRP-DC-RMS	SRP-DC-COSH	MVDR-LP	MVDR-IS	MVDR-RMS	MVDR-COSH
20–675	50.2	53.8	57.5	47.1	52.4	51.3	59.4	55.7	45.4	62.0	52.0	53.5
